# Cognition Moderates the Relationship Between Hearing and Mobility in Cognitively Normal Older Adults

**DOI:** 10.1093/geroni/igab046.618

**Published:** 2021-12-17

**Authors:** Daniel Pupo, Brent Small, Jennifer Deal, Nicole Armstrong, Susan Resnick, Frank Lin, Luigi Ferrucci, Qu Tian

**Affiliations:** 1 University of South Florida, University of South Florida, Florida, United States; 2 University of South Florida, Tampa, Florida, United States; 3 Johns Hopkins University, Baltimore, Maryland, United States; 4 Warren Alpert Medical School of Brown University, Providence, Rhode Island, United States; 5 National Institute on Aging, Baltimore, Maryland, United States; 6 Johns Hopkins University, Johns Hopkins University, Maryland, United States

## Abstract

Recent data has shown a consistent but modest association between hearing impairment and poor mobility; both are strongly associated with cognition. Cognitive function may moderate the relationship between hearing and mobility. We analyzed 601 cognitively normal older participants from the Baltimore Longitudinal Study of Aging who had concurrent data on cognition (attention, executive function, sensorimotor function), hearing (pure-tone average, PTA), and mobility (6-meter gait speed, 400-meter time). We performed multivariable-adjusted linear regression to test two-way interactions between each cognitive measure and PTA. There were significant PTA interactions with all cognitive measures on 400-meter time. There was a significant interaction between PTA and sensorimotor function on 6-meter gait speed. Among cognitively normal older adults, poorer hearing is more strongly associated with poor mobility in those with low cognition, especially sensorimotor function. Future studies are needed to understand how cognition may moderate the relationship of hearing impairment with mobility decline over time.

